# The effects of ductal size on the severity of pulmonary hypertension in children with patent ductus arteriosus (PDA): a multi-center study

**DOI:** 10.1186/s12890-021-01449-y

**Published:** 2021-03-05

**Authors:** Josephat M. Chinawa, Bartholomew F. Chukwu, Awoere T. Chinawa, Chika O. Duru

**Affiliations:** 1 Pediatric Cardiologist, College of Medicine, Department of Pediatrics, University of Nigeria/University of Nigeria Teaching Hospital (UNTH), Ituku-Ozalla, Enugu State Nigeria; 2 Department of Pediatrics, University of Nigeria/University of Nigeria Teaching Hospital (UNTH), Enugu State, Ituku-Ozalla, Nigeria; 3 Enugu State University Teaching Hospital, Enugu, Enugu State Nigeria; 4 Department of Paediatrics and Child Health, Niger Delta University Teaching Hospital, Okolobiri, Bayelsa State Nigeria

**Keywords:** PDA, Ductal size, Pulmonary hypertension, Children

## Abstract

**Introduction:**

Patent ductus arteriosus (PDA) is a common acyanotic heart disease that presents with variable symptoms.

**Objectives:**

This study is therefore aimed at determining the relationship between gender, age, and size of PDA and pulmonary hypertension. This study also seeks to determine the prevalence of elevated pulmonary artery systolic pressure in children with PDA.

**Patients and methods:**

A descriptive study of children with patent ductus arteriosus was carried out from 2016 to 2020 in three institutions. The data were analysed with the IBM SPSS statistics for windows, version 20 (IBM Corp, Chicago)

**Result:**

The mean ductal size was 3.78 (2.39) mm, with a minimum of 1.0 mm and a maximum size of 10.0 mm. The mean ductal size for males, 4.02 (2.53) mm was comparable with that of the females, 3.61 (2.28) mm (Student T-test = 0.8, 0.4). The mean pulmonary artery systolic pressure (PASP) of the patients was 43.36 (24.46) mmHg. Also the mean PASP was comparable among the males and the females, 48.37 (26.69) mmHg versus 39.63 (22.16) mmHg (Student T-test = 1.81, p = 0.07). There was no correlation between age and PASP (correlation coefficient = 0.009, p = 0.92). Sixty point two percent (60.2%) (62/103) of children with PDA had pulmonary hypertension. The proportion of males with pulmonary hypertension, 48.39% (30/62) was comparable with that of the females, 51.61% (32/62) (Chi^2^ = 2.05, p = 0.15) and females are 1.8 times more likely to have pulmonary hypertension as males (odds ratio 1.81, 95% CI 0.8–4.1). There was a positive correlation between ductal size and PASP (Pearson correlation coefficient = 0.26, p value = 0.007). Those with moderate and large sized duct tend to have moderate and severe pulmonary hypertension respectively and this is statistically significant. Chi^2^ = 17.85, p = 0.007

**Conclusion:**

The prevalence of pulmonary hypertension in children with PDA is 60.2%. Moderate and large size duct presents with moderate and severe pulmonary hypertension respectively. Females are 1.8 times more likely to have pulmonary hypertension than the males.

**Supplementary Information:**

The online version contains supplementary material available at 10.1186/s12890-021-01449-y.

## Introduction

The ductus arteriosus is a remnant of the distal sixth aortic arch. Patent ductus arteriosus (PDA) is usually a left aortic remnant [1]. It could be right-sided or even bilateral.

The prevalence of PDA is variable ranging from [5 to 10%] [1, 2]. This lesion is the major cause of mortality and morbidity in infancy. If not closed, it could lead to pulmonary hypertension later in life [3]. The hemodynamic effects of PDA are well known including pulmonary hypertension, systemic hypo-perfusion, and volume overload on the left ventricle [4].

In children with isolated PDA, shunt volume and pulmonary hypertension are determined by the size of the open communication and the pulmonary vascular resistance (PVR) [5]. Pulmonary overflow results in decreased pulmonary compliance and the magnitude of the excess pulmonary blood flow depends on some factors [1]. The larger the internal diameter of the most-narrow portion of the ductus arteriosus, the larger the left-to-right shunt, and thus the higher the chance of pulmonary hypertension. If the ductus arteriosus is small or narrow, the magnitude of the shunt will also be affected [1, 6]. Furthermore, the magnitude of the left-to-right shunt is partially controlled by the relationship of PVR to the systemic vascular resistance (SVR). If the SVR is high and/or the PVR is low, the flow through the ductus arteriosus is potentially large [6].

Age is an important predictor of pulmonary hypertension and pulmonary vascular disease (PVD). It has been postulated that PDA may have a more marked effect on the pulmonary circulation than a ventricular septal defect and that irreversible pulmonary vascular changes may occur under two years of age [7]. This could be explained by the high-pressure pulsatile flow transmitted from the aorta to the pulmonary artery throughout the cardiac cycle in PDA.

It is very pertinent to note that echocardiographic assessment of right ventricular function and measurements of peak velocities of valvar regurgitations are the two major approaches that predict pulmonary artery systolic pressure measurements of PDA [8, 9]. Granted that the right-sided heart catheterization is very crucial in assessing pulmonary artery pressure in children especially for operability, however, it is obvious that performing catheterization in all patients with heart disease will be expensive, impractical, and with its attendant risk factors [8–11]. Echocardiographic assessment of pulmonary artery pressure in children with PDA is therefore very simple, accurate, and safe with a very high cost–benefit ratio [8–10].

Assessment of pulmonary artery systolic pressure in children with PDA is very crucial in this setting, as it will help in risk categorization and timely initiation of management. A careful search in the literature showed that there is no single study in Nigeria on this topic.

No known study in this vicinity looked at any link between the size of PDA and pulmonary hypertension. This study is therefore aimed at determining the relationship between gender, age, and size of PDA and pulmonary hypertension. This study also seeks to determine the prevalence of elevated pulmonary artery systolic pressure in children with PDA.

## Materials and methods

### Study area

This is an observational cross-sectional study, carried out in one private and two public hospitals namely the University of Nigeria Teaching Hospital, Blessed children hospital and Niger Delta University, over a-six-year period from 2016 to 2020.

### Study population

These were children aged one month to 14 years who attended the paediatric cardiology clinics of the three hospitals of study.

### Study design

This was an observational cross-sectional study conducted in three institutions from the year 2016 to 2020.

### Inclusion and exclusion criteria

Children with PDA aged one month to 14 years and who gave consent or assent were included in the study while children whose parents did not give verbal consent and children with previously corrected congenital heart disease were excluded from the study. Preterm infants and infants less than 1-month old were also excluded from the study.

### Consent

Verbal consent for echocardiography was obtained from each parent/caregiver of the subjects from the time they presented to the clinic for cardiac evaluation.

### Child assent

This was obtained in children who were older than seven years.

The study had a quality control where other cardiologist made a diagnosis of congenital heart disease at different intervals to reduce bias.

### Definition of Patent ductus arteriosus using echocardiography

The best view for measuring the size of the PDA is the ductal view. This is otherwise called the high left parasternal short-axis view. Paying attention to the main pulmonary artery, the origin of the right pulmonary artery and the left pulmonary artery can be seen, the PDA could be visualized at the left. The size of the PDA was taken at the narrowest point, before it entered into the main pulmonary artery [12].

### Assessment of size of patent ductus arteriosus

In this research work, insignificant or small size PDA was taken as the diameter of patent ductus arteriosus of 1–1.5 mm, moderate size PDA was taken as the diameter of patent ductus arteriosus of 1.5-3 mm while large size PDA is taken as the diameter of patent ductus arteriosus of more than and equal to 3 mm [13].

### Assessment of pulmonary hypertension

At the 6th World Symposium on Pulmonary Hypertension (PH), the mean pulmonary artery systolic pressure(mPAP) the threshold for pulmonary hypertension which was formerly ≥ 25 mmHg has been changed to mean pulmonary artery systolic pressure(mPAP) > 20 mmHg [14]. The rationale for this change is that the threshold ≥ 25 mmHg is determined by individual preference or convenience, whereas the new threshold is based on scientific evidence. This change may facilitate earlier PH detection, risk stratification and screening programmes [14].

Pulmonary hypertension was calculated in this study by summing the calculated pressure gradient over the tricuspid valve and right atrial pressure.

Normal right atrial pressure of 3 mmHg is usually added to TR velocity when the IVC is normal in size and collapsible, high right atrial pressure of 15 mmHg is added to TR Velocity when the IVC is dilated and does not collapse normally. However, intermediate values of 8 mmHg will be added to TR velocity in any scenario that does not meet the normal and high values. We used the third criteria where RA pressure of 8 mmHg was added to TR velocity to get pulmonary artery systolic pressure. According to current guidelines by Chemla et al. [15], pulmonary hypertension is defined if mean pulmonary artery pressure is above 20 mmHg. Mean pulmonary artery pressure of 20 mmHg corresponds to pulmonary artery systolic pressure of about 30 mmHg. So in this study, pulmonary artery systolic pressure of 30 mmHg was taken as pulmonary hypertension [15]. Pulmonary hypertension was classified as mild = 30–50 mmhg; moderate = 51–65 mmhg and severe≥ 65 mmHg [16].

Children who had PDA were examined in a supine, left lateral decubitus position. The views of PDA were obtained in subcostal, left parasternal, apical, and suprasternal windows [17].

We used the Bernoulli equation to estimate the pulmonary arterial systolic pressure (PASP = 4 V2 + right atrial pressure) where V indicates peak systolic velocity of the regurgitant jet as far as pulmonary stenosis is ruled out [13, 14].

### Statistical analysis

The data were analysed with the IBM SPSS statistics for windows, version 20 (IBM Corp, Chicago). Categorical variables were analysed in form of proportions and percentages and presented in form of tables. Continuous variables including ductal size and pulmonary artery pressure were analysed and summarized as means and standard deviations. Means of continuous variables were compared with the Student T- test while categorical variables were compared with Chi-square. Correlation between continuous variables was determined using Pearson correlation. Gender probability of having pulmonary hypertension was reported using adjusted odds ratios (AOR) and 95% confidence interval. Test of significance was set at p < 0.05.

## Result

### Demography

One hundred and four (104) patients aged one month to 14 years were evaluated for the effect of ductal size on the severity of pulmonary artery systolic pressure (PASP) among children with patent ductus arteriosus (PDA). The males comprised 42.3% (44/104). The mean age of the patients was 22.72 (35.46) months. Pulmonary hypertension was more in infants than older children, 54.5% versus 45.5%, although the difference was not statistically significant (χ^2^ = 0.3, p = 0.6), (odd ratio = 0.8, 95% confidence interval; 0.3–1.9).

### Ductal size and pulmonary artery systolic pressure

The mean ductal size was 3.78 (2.39) mm, with a minimum of 1.0 mm and a maximum size of 10.0 mm. The mean ductal size for males, 4.02 (2.53) mm was comparable with that of the females, 3.61 (2.28) mm (Student T-test = 0.8, 0.4). We classified ductal size into small, moderate and large and Table 1 illustrates the frequency of occurrence of the different sizes.

**Table 1:** Frequency of different classes of ductal size

Class of ductal size	Frequency	Percent
Small	22	21.4
Moderate	31	30.1
Large	50	48.5
Total	103^a^	100.0

^a^One patient did not have data for ductal size

The mean PASP of the patients was 43.36 (24.46) mmHg. Also the mean PASP was comparable among the males and the females, 48.37 (26.69) mmHg versus 39.63 (22.16) mmHg (Student T-test = 1.81, p = 0.07). There was no correlation between age and PASP (correlation coefficient = 0.009, p = 0.92).

### Pulmonary hypertension

PASP of 30 mmHg and above was regarded as pulmonary hypertension and it was observed that 60.2% (62/103) had pulmonary hypertension. The proportion of males with pulmonary hypertension, 48.39% (30/62) was comparable with that of the females, 51.61% (32/62) (χ^2^ = 2.05, p = 0.15), although the females are 1.8 times more likely to have pulmonary hypertension as the males (odds ratio 1.81, 95% CI 0.8–4.1).

The severity of pulmonary hypertension was also classified into mild, moderate and severe and the frequency of different severities shown in Table 2.

**Table 2:** Frequency of different severities of pulmonary hypertension

Severity	Frequency	Percent
Mild	22	35.48
Moderate	19	30.65
Severe	21	33.87
Total	62	100.0

The relationship between ductal size and severity of pulmonary hypertension is shown in Table 3 which revealed that 42.86% of those with small ductal size had mild pulmonary hypertension and none had severe pulmonary hypertension while 44.44% of those with large ductal size had mild hypertension and 33.33% had severe pulmonary hypertension. Those with moderate and large-sized duct tend to have moderate and severe pulmonary hypertension respectively and this is statistically significant. χ^2^ = 17.85, p = 0.007.

**Table 3:** Relationship between the class of ductal size and severity of pulmonary hypertension

Class of ductal size	Severity of pulmonary hypertension	Total n (%)		
		Mild n(%)	Moderate n(%)	Severe n(ˀ%)
Small	3 (42.86)	4 (57.14)	0 (0)	7 (100)
Moderate	3 (15.79)	7 (36.84)	9 (47.37)	19 (100)
Large	16 (44.44)	8 (22.22)	12 (33.33)	36 (100)
Total	22 (35.48)	19 (30.65)	21 (33.87)	62 (100)

Chi-square = 17.85, p = 0.007

### Correlation between size of PDA and PASP

**Fig. 1:**
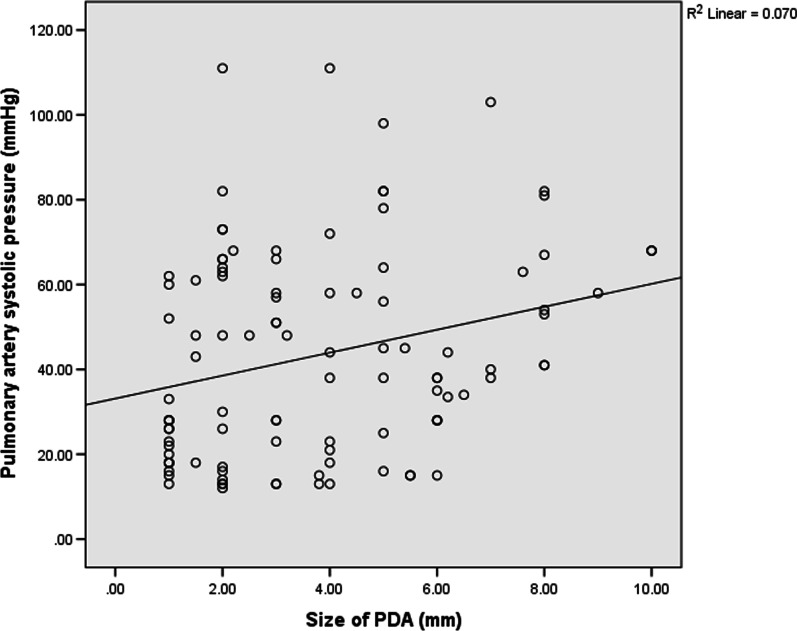
Correlation between pulmonary artery systolic pressure and size of PDA

**Fig. 2:**
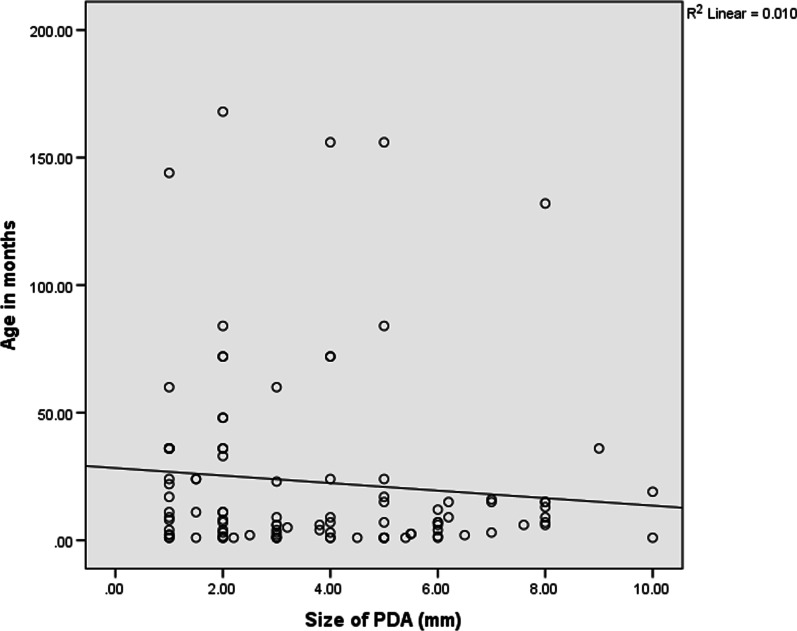
Correlation between age of patients and size of PDA

There was a positive correlation between ductal size and PASP as illustrated in Fig. 1 (Pearson correlation coefficient = 0.26, p value = 0.007). As the ductal size increases, the PASP also increases. There was no correlation between age of patients and size of PDA as shown in Fig. 2, (correlation coefficient = − 0.10, p = 0.32).

### Prevalence of PDA

Seven hundred and fifty-eight (758) echocardiography reports on children with cardiac disease were studied and a diagnosis of PDA was in 104 children giving a prevalence of 13.9%.

## Discussion

Pulmonary artery systolic pressure is an integer and harbinger of pulmonary hypertension [1]. Pulmonary artery systolic pressure is a mirror image of right heart haemodynamic, and is very important in the diagnosis of pulmonary hypertension. Pulmonary hypertension has been noted as the fourth most prevalent cardiovascular disease in the world, though this goes unnoticed by the WHO Global Action Plan and Global Burden of Diseases [2]. Data on the world prevalence of pulmonary hypertension in children with PDA is lacking; a well-knitted and coordinated action is therefore needed for screening and intervention [3].

The prevalence of pulmonary hypertension in children with PDA in this study is 60.2% and is more with a large ductal size. Progressive hypertension, intimal proliferation and prolonged hypoxaemia could explain this phenomenon [18–20]. Weijerman et al. [21] noted a prevalence of 5.8% but this was mainly among termed neonates. Our study transcends neonatal age. Studies on the prevalence of pulmonary hypertension were mainly among children with congenital heart disease and there is a paucity of studies in children with PDA. The available prevalence of pulmonary hypertension was focused on preterm babies.

It is evidenced from this study, that children with moderate and large-sized duct tend to have moderate and severe pulmonary hypertension respectively. Studies have shown that increase blood volume that shunts to the right heart towards the lung from a large patent PDA and laxity of the vasa-vasorum in the duct could all account for this high risk of pulmonary hypertension among children with large sized PDA [12]. Niu et al. [22], also noted that large PDA could result in severe pulmonary hypertension and irreversible pulmonary vascular disease. They realized that when this large PDA was closed with a catheter, that right ventricular failure usually ensues. In a large ductus arteriosus, there is build up in pulmonary artery pressure with resultant turbulent flow across the defect. With this continued shunt, coupled with neo-vascularization and intimal proliferation, there could be a shunt reversal and pulmonary vascular disease [23].

Though age at presentation has been noted not to correlate with pulmonary hypertension, however, infants have more odds and are seen to present with pulmonary hypertension than older children. This is quite contrary to other studies where children less than 1 year of age are unlikely to present with features of pulmonary arterial hypertension [7, 24–27].

The increased episodes of pulmonary hypertension among older children as seen in other studies, could be due to the fact that high-pressure and pulsatile flow from the aorta to the pulmonary artery, if not arrested by closure or ligation, could lead to progression of vascular disease (PVD), with increased pulmonary artery pressure more than systemic pressure, there could be a shunt reversal, leading to eisenmenger syndrome [7, 26–29]. There is general agreement that patients with eisenmenger syndrome should not have the defect closed and in fact, the defect can be beneficial because the defect acts as an outlet for the right ventricle (RV) to pump into the systemic circulation and maintain cardiac output at the cost of arterial desaturation [23–25, 30, 31].

This study reveals that female children with PDA are 1.8 times more likely to have pulmonary hypertension than the male counterparts. This finding is documented by other researchers who also noted a female preponderance in children with elevated pulmonary pressures [32–34]. The theory of “sex paradox” elaborated in pulmonary hypertension has long been known in females. They have a higher susceptibility than males with a female-to-male ratio is 4:1 [33]. Genetic factors have been linked to this increased risk in female children. A genetic mutation in the bone morphogenetic protein 2 (BMPR2) gene has been linked to pulmonary arterial hypertension. Females children are more likely to express the mutation in the BMPR2 receptor [33].

Several studies have investigated the link between BMPR2 and oestrogen signalling, which has been proposed as a very important trigger for female predominance. For instance, Austin et al. [37] noted that when oestrogen receptor alpha binds to the BMPR2 promoter, its gene expression was remarkably reduced in females [33]. Similarly, recent studies have noted that sex chromosomes as another reason for females to have a higher likelihood for pulmonary hypertension. Yan et al. [35] demonstrated that SRY on the Y chromosome regulates and binds to the BMPR2 receptor and promoter to reduce the prevalence of PAH in males [33].

The World Health Organization (WHO) Group 1 for pulmonary hypertension and other studies noted that the female predominance could be related to differences in their immune system [34–37].

The prevalence of Patent ductus arteriosus noted in this study is 13.9%. This prevalence rate is different from that obtained in the US which showed an estimated prevalence of between 0.02% and 0.006% of babies born at term [38]. However, the reported prevalence of PDA in term neonates is 1 in 2,000 births, accounting for 5%–10% of all congenital heart disease [6].

## Conclusion

The prevalence of pulmonary hypertension in children with PDA is 60.2%. Moderate and large size duct presents with moderate and severe pulmonary hypertension respectively. Females are 1.8 times more likely to have pulmonary hypertension than the males.

### Recommendations

Early closure or ligation PDA is recommended to avert the development of pulmonary hypertension in children especially those with large PDA (Additional file 1).

### Strength of the study

This is the first time this work is done in the vicinity and can form a template for other studies. In addition, its multi -enter nature gives it an added advantage.

## Limitations

Sample size is relatively not large.

## Supplementary Information

**Additional file 1.** Additional file containg the raw data of SPSS.

## Data Availability

All data generated or analysed during this study are included in this article [and its supplementary information files].
