# Supplementary material for: The effects of ductal size on the severity of pulmonary hypertension in children with patent ductus arteriosus (PDA): a multi-center study
Source: BMC Pulm Med. 2021 Mar 5;21:79. doi: 10.1186/s12890-021-01449-y (PMC7934417; doi:10.1186/s12890-021-01449-y)

PDA SPSS

| **Sex** | | | | | |
| --- | --- | --- | --- | --- | --- |
|  | | Frequency | Percent | Valid Percent | Cumulative Percent |
| Valid | male | 44 | 42.3 | 42.3 | 42.3 |
|  | female | 60 | 57.7 | 57.7 | 100.0 |
|  | Total | 104 | 100.0 | 100.0 |  |

| **Descriptive Statistics** | | | | | |
| --- | --- | --- | --- | --- | --- |
|  | N | Minimum | Maximum | Mean | Std. Deviation |
| Age in months | 103 | 1.00 | 168.00 | 22.7209 | 35.45883 |
| Valid N (listwise) | 103 |  |  |  |  |

| **Descriptive Statistics** | | | | | |
| --- | --- | --- | --- | --- | --- |
|  | N | Minimum | Maximum | Mean | Std. Deviation |
| Size of PDA (mm) | 103 | 1.00 | 10.00 | 3.7854 | 2.38679 |
| Valid N (listwise) | 103 |  |  |  |  |

| **Group Statistics** | | | | | |
| --- | --- | --- | --- | --- | --- |
|  | Sex | N | Mean | Std. Deviation | Std. Error Mean |
| Size of PDA (mm) | male | 44 | 4.0159 | 2.52908 | .38127 |
|  | female | 59 | 3.6136 | 2.28167 | .29705 |

| **Independent Samples Test** | | | | | | | | | | |
| --- | --- | --- | --- | --- | --- | --- | --- | --- | --- | --- |
|  | | Levene's Test for Equality of Variances | | t-test for Equality of Means | | | | | | |
|  |  | F | Sig. | t | df | Sig. (2-tailed) | Mean Difference | Std. Error Difference | 95% Confidence Interval of the Difference | |
|  |  |  |  |  |  |  |  |  | Lower | Upper |
| Size of PDA (mm) | Equal variances assumed | .073 | .787 | .845 | 101 | .400 | .40235 | .47609 | -.54208 | 1.34678 |
|  | Equal variances not assumed |  |  | .832 | 87.220 | .407 | .40235 | .48333 | -.55829 | 1.36298 |

| **Ductal size** | | | | | |
| --- | --- | --- | --- | --- | --- |
|  | | Frequency | Percent | Valid Percent | Cumulative Percent |
| Valid | small | 22 | 21.4 | 21.4 | 21.4 |
|  | moderate | 31 | 30.1 | 30.1 | 51.5 |
|  | large | 50 | 48.5 | 48.5 | 100.0 |
|  | Total | 103 | 100.0 | 100.0 |  |

| **Descriptive Statistics** | | | | | |
| --- | --- | --- | --- | --- | --- |
|  | N | Minimum | Maximum | Mean | Std. Deviation |
| New pulmonary pressure | 103 | 12.00 | 111.00 | 43.3641 | 24.46278 |
| Valid N (listwise) | 103 |  |  |  |  |

| **Group Statistics** | | | | | |
| --- | --- | --- | --- | --- | --- |
|  | Sex | N | Mean | Std. Deviation | Std. Error Mean |
| New pulmonary pressure | male | 44 | 48.3750 | 26.69163 | 4.02392 |
|  | female | 59 | 39.6271 | 22.15766 | 2.88468 |

| **Independent Samples Test** | | | | | | | | | | |
| --- | --- | --- | --- | --- | --- | --- | --- | --- | --- | --- |
|  | | Levene's Test for Equality of Variances | | t-test for Equality of Means | | | | | | |
|  |  | F | Sig. | t | df | Sig. (2-tailed) | Mean Difference | Std. Error Difference | 95% Confidence Interval of the Difference | |
|  |  |  |  |  |  |  |  |  | Lower | Upper |
| New pulmonary pressure | Equal variances assumed | 1.394 | .240 | 1.815 | 101 | .072 | 8.74788 | 4.81880 | -.81133 | 18.30709 |
|  | Equal variances not assumed |  |  | 1.767 | 82.416 | .081 | 8.74788 | 4.95109 | -1.10067 | 18.59643 |

| **Correlations** | | | |
| --- | --- | --- | --- |
|  | | Age in months | New pulmonary pressure |
| Age in months | Pearson Correlation | 1 | .009 |
|  | Sig. (2-tailed) |  | .925 |
|  | N | 103 | 103 |
| New pulmonary pressure | Pearson Correlation | .009 | 1 |
|  | Sig. (2-tailed) | .925 |  |
|  | N | 103 | 103 |

| **New pulmonary hypertension** | | | | | |
| --- | --- | --- | --- | --- | --- |
|  | | Frequency | Percent | Valid Percent | Cumulative Percent |
| Valid | yes | 62 | 60.2 | 60.2 | 60.2 |
|  | no | 41 | 39.8 | 39.8 | 100.0 |
|  | Total | 103 | 100.0 | 100.0 |  |

| **Sex * New pulmnary hypertension Crosstabulation** | | | | |
| --- | --- | --- | --- | --- |
| Count | | | | |
|  | | New pulmnary hypertension | | Total |
|  |  | yes | no |  |
| Sex | male | 30 | 14 | 44 |
|  | female | 32 | 27 | 59 |
| Total | | 62 | 41 | 103 |

| **Risk Estimate** | | | |
| --- | --- | --- | --- |
|  | Value | 95% Confidence Interval | |
|  |  | Lower | Upper |
| Odds Ratio for Sex (male / female) | 1.808 | .800 | 4.086 |
| For cohort New pulmnary hypertension = yes | 1.257 | .923 | 1.713 |
| For cohort New pulmnary hypertension = no | .695 | .416 | 1.163 |
| N of Valid Cases | 103 |  |  |

| **Ductal size** | | | | | |
| --- | --- | --- | --- | --- | --- |
|  | | Frequency | Percent | Valid Percent | Cumulative Percent |
| Valid | small | 22 | 21.4 | 21.4 | 21.4 |
|  | moderate | 31 | 30.1 | 30.1 | 51.5 |
|  | large | 50 | 48.5 | 48.5 | 100.0 |
|  | Total | 103 | 100.0 | 100.0 |  |

| **New severity of pulmonary hypertension** | | | | | |
| --- | --- | --- | --- | --- | --- |
|  | | Frequency | Percent | Valid Percent | Cumulative Percent |
| Valid |  | 41 | 39.8 | 39.8 | 39.8 |
|  | mild | 22 | 21.4 | 21.4 | 61.2 |
|  | moderate | 19 | 18.4 | 18.4 | 79.6 |
|  | severe | 21 | 20.4 | 20.4 | 100.0 |
|  | Total | 103 | 100.0 | 100.0 |  |

| **Ductal size * New severity of pulmonary hypertension Crosstabulation** | | | | | | |
| --- | --- | --- | --- | --- | --- | --- |
| Count | | | | | | |
|  | | New severity of pulmonary hypertension | | | | Total |
|  |  |  | mild | moderate | severe |  |
| Ductal size | small | 15 | 3 | 4 | 0 | 22 |
|  | moderate | 12 | 3 | 7 | 9 | 31 |
|  | large | 14 | 16 | 8 | 12 | 50 |
| Total | | 41 | 22 | 19 | 21 | 103 |

| **Chi-Square Tests** | | | |
| --- | --- | --- | --- |
|  | Value | df | Asymp. Sig. (2-sided) |
| Pearson Chi-Square | 17.847^a^ | 6 | .007 |
| Likelihood Ratio | 21.832 | 6 | .001 |
| N of Valid Cases | 103 |  |  |
| a. 3 cells (25.0%) have expected count less than 5. The minimum expected count is 4.06. | | | |

| **Correlations** | | | |
| --- | --- | --- | --- |
|  | | Size of PDA (mm) | New pulmonary pressure |
| Size of PDA (mm) | Pearson Correlation | 1 | .264^**^ |
|  | Sig. (2-tailed) |  | .007 |
|  | N | 103 | 103 |
| New pulmonary pressure | Pearson Correlation | .264^**^ | 1 |
|  | Sig. (2-tailed) | .007 |  |
|  | N | 103 | 103 |
| **. Correlation is significant at the 0.01 level (2-tailed). | | | |

| **Correlations** | | | |
| --- | --- | --- | --- |
|  | | Age in months | Size of PDA (mm) |
| Age in months | Pearson Correlation | 1 | -.099 |
|  | Sig. (2-tailed) |  | .319 |
|  | N | 103 | 103 |
| Size of PDA (mm) | Pearson Correlation | -.099 | 1 |
|  | Sig. (2-tailed) | .319 |  |
|  | N | 103 | 103 |


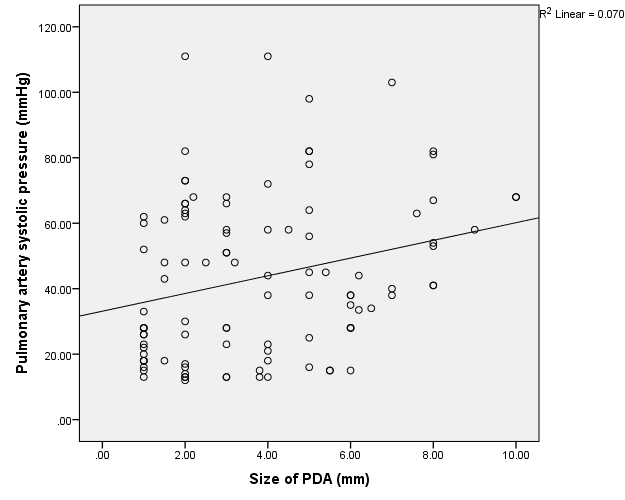


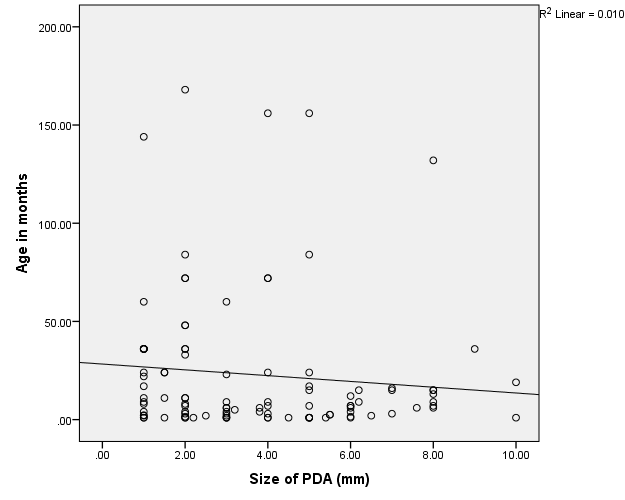

Supplement: Supplementary file 1 — Additional file 1. Additional file containg the raw data of SPSS. [file 12890_2021_1449_MOESM1_ESM.docx]
